# Elucidating the origin of HLA-B*73 allelic lineage: Did modern humans benefit by archaic introgression?

**DOI:** 10.1007/s00251-016-0952-8

**Published:** 2016-09-30

**Authors:** Yoshiki Yasukochi, Jun Ohashi

**Affiliations:** 1 Department of Human Functional Genomics, Life Science Research Center, Mie University, 1577 Kurima-machiya, Tsu, Mie 514-8507 Japan; 2 Department of Biological Sciences, Graduate School of Science, The University of Tokyo, 7-3-1 Hongo, Bunkyo-ku, Tokyo, 113-0033 Japan

**Keywords:** Allelic divergence, Denisovan, *HLA*, HLA-B*73, Introgression, Neanderthal

## Abstract

A previous study reported that some of the human leukocyte antigen (*HLA*) alleles and haplotypes in present-day humans were acquired by admixture with archaic humans; specifically, an exceptionally diverged HLA-B*73 allele was proposed to be transmitted from Denisovans, although the DNA sequence of HLA-B*73 has not been detected in the Denisovan genome. Here, we argue against the hypothesis that HLA-B*73 introgressed from Denisovans into early modern humans. A phylogenetic analysis revealed that HLA-B*73:01 formed a monophyletic group with a chimpanzee *MHC-B* allele, strongly suggesting that the HLA-B*73 allelic lineage has been maintained in humans as well as in chimpanzees since the divergence of humans and chimpanzees. The global distribution of HLA-B*73 allele showed that the population frequency of HLA-B*73 in west Asia (0.24 %)—a possible site of admixture with Denisovans—is lower than that in Europe (0.72 %) and in south Asia (0.69 %). Furthermore, HLA-B*73 is not observed in Melanesia even though the Melanesian genome contains the highest proportion of Denisovan ancestry in present-day human populations. Single nucleotide polymorphisms in HLA-A*11-HLA-C*12:02 or HLA-A*11-C*15 haplotypes, one of which was assumed to be transmitted together with HLA-B*73 from Denisovans by the study of Abi-Rached and colleagues, were not differentiated from those in other *HLA-A-C* haplotypes in modern humans. These results do not support the introgression hypothesis. Thus, we conclude that it is highly likely that HLA-B*73 allelic lineage has been maintained in the direct ancestors of modern humans.

**Electronic supplementary material:**

The online version of this article (doi:10.1007/s00251-016-0952-8) contains supplementary material, which is available to authorized users.

Large-scale comparisons of whole genome sequences of Neanderthals and Denisovan with those of present-day humans have revealed that admixture occurred between archaic and non-African modern humans (Green et al. 2010; Reich et al. 2010; Meyer et al. 2012; Prüfer et al. 2014). The identification of the characteristic signature of adaptive introgression in the genome sequences of modern humans has been of particular interest. A study on the human leukocyte antigen (*HLA*, called major histocompatibility complex (*MHC*) in vertebrates) class I region of two archaic human genomes proposed that modern humans acquired the HLA-B*73 allele via admixture with archaic humans (Abi-Rached et al. 2011). Abi-Rached et al. (2011) assumed that the B*73 allelic lineage was translocated to the extant *HLA-B* locus (*MHC-BI*) from the distinct fictive *HLA-B* locus (*MHC-BII*) by interlocus recombination before the split of Homininae at the latest. They concluded that the ancestral population of present-day humans lost the HLA-B*73 allelic lineage after the divergence of modern humans and Denisovans, and subsequently this allele was re-introduced in modern humans through the admixture with the archaic humans. The introgression hypothesis of *HLA* class I gene seems to provide a plausible explanation on why the HLA-B*73 found in present-day humans is structurally divergent from other *HLA-B* alleles. However, the introgression hypothesis does not appear to be well grounded. First, in contrast to other studies that showed signatures of adaptive introgression from Neanderthals or Denisovans (Racimo et al. 2015), the actual introgressed segment or haplotype (i.e., HLA-B*73) has not been detected in the genome of archaic humans in the study conducted by Abi-Rached et al. (2011). Second, HLA-B*73:01—a putative allele derived from the archaic HLA-B*73—is observed in Africans as well as non-Africans. Based on the computer simulation, Abi-Rached et al. (2011) mentioned that the presence of HLA-B*73:01 in Africa was due to back migrations. However, this explanation negates an implicit assumption that introgressed haplotypes from Denisovans or Neanderthals are not shared with Africans. Third, to our knowledge, HLA-B*73 has not been detected in Melanesians, who have derived 4∼6 % of their genome from Denisovans (Reich et al. 2010). Fourth, although the 5′ and 3′ flanking regions of HLA-B*73:01 show similarity with other *HLA-B* alleles, it does not provide any information on the occurrence of introgression, simply because recombination can occur regardless of the introgression. Lastly, in the study conducted by Abi-Rached et al. (2011), a computer simulation assuming neutrality at the *HLA-B* locus was used to provide support for the introgression hypothesis; however, it is well known that the classical class I *HLA* loci have been subjected to strong balancing selection (Satta et al. 1994). A neutral model cannot reproduce the coalescent process of *HLA* alleles that have been affected by strong balancing selection (Takahata 1990). Therefore, the origin of archaic-like *HLA* alleles and haplotypes in modern humans remains a matter of debate. In the present study, we discuss a possibility that the HLA-B*73 lineage has been actually maintained over the course of the evolution of modern humans.

To investigate the molecular evolution of human HLA-B*73, we examined phylogenetic relationships among Homininae *MHC-B* genomic sequences (ca. 8 kb). For phylogenetic analysis, nucleotide sequences at the *MHC-B* locus in human and three non-human primates—common chimpanzee (*Pan troglodytes*, *Patr-B*), pygmy chimpanzee (*Pan paniscus*, *Papa-B*), and western gorilla (*Gorilla gorilla*, *Gogo-B*)—were obtained from the NCBI database (http://www.ncbi.nlm.nih.gov/) after removing relatively short nucleotide sequences. Of the three HLA-B*73 alleles deposited in the database, HLA-B*73:02 (GenBank accession number AY040668) and HLA-B*73var (GenBank accession number HM347714) alleles were removed from analyses due to insufficient information on the frequency and relatively long undetermined sequences. Nucleotide sequences were aligned by using the MUSCLE algorithm in MEGA v.5.2 (Tamura et al. 2011) with the complete-deletion option (insertions and deletions were not considered). Then, a maximum likelihood (ML) tree was generated based on the Kimura two-parameter model (Kimura 1980) with discrete gamma distribution among sites using nucleotide substitutions among 23 Homininae *MHC-B* genomic sequences composed of 8 *HLA-B*, 9 *Patr-B*, 1 *Papa-B*, and 5 *Gogo-B* (Online Resource 1). The constructed phylogenetic tree displayed that the HLA-B*73:01 allele formed a clade with Patr-B*17:01, distinct from the other *HLA-B* and *Patr-B* alleles, and at least four *MHC-B* alleles (HLA-B*73, Patr-B*17, Gogo-B*06, and Gogo-B*07) appeared to be a member of the distinct *MHC-B* allelic lineage in Homininae. It is important to note that the Patr-B*17 has at least three subordinate alleles, Patr-B*17:01, Patr-B*17:02, and Patr-B*17:03. Similar to the designation given by Abi-Rached et al. (2011), we refer to the HLA-B*73 and Patr-B*17 alleles as *MHC-BII* and refer to the other *MHC-B* alleles (with the exception of two *Gogo-B* alleles mentioned earlier, Gogo-B*06 and Gogo-B*07) as *MHC-BI*. The Gogo-B*06 and Gogo-B*07 alleles were more distant from *MHC-BI* alleles than *MHC-BII* alleles (Online Resource 1). The *MHC-BII* alleles shared *BII*-specific mutations that were not observed in *MHC-BI* allelic lineages (nucleotides shown in red in Online Resource 2), and the pairwise mean genetic distance (measured by Kimura two-parameter model with discrete gamma distribution among sites) between *MHC-BI* and *BII* alleles in the chimpanzee (0.04 ± 0.001) was significantly larger than that between the same alleles in the human (0.03 ± 0.001) (*P* value < 0.05 by Welch’s *t* test), supporting the presence of both, *BI* and *BII*, allelic lineages in chimpanzees. The presence of both *BI* and *BII* allelic lineages in chimpanzees demonstrated by the phylogenetic analysis and the pairwise mean genetic distance in the present study indicates that the exceptionally divergent HLA-B*73 cannot be only explained through admixture with archaic humans. Therefore, it is reasonable to expect that the B*73 allelic lineage has also been maintained in modern humans. A fundamental issue associated with the introgression hypothesis suggested by Abi-Rached et al. (2011) is that it is not clear why the HLA-B*73 allele in the Denisovan genome has not been homogenized to *MHC-BI* sequences by inter-allelic recombination during the archaic human evolution. Although the origin of the *MHC-BII* allelic lineage remains unclear, it is likely that recombination between *MHC-BII* and *MHC-BI* allelic lineages may have seldom occurred due to their dissimilar sequences or perhaps balancing selection may have maintained two distinct allelic lineages.

Next, we discuss whether the HLA-B*73 allele and some *HLA* class I alleles/haplotypes in present-day humans have been acquired through interbreeding with archaic humans, on the basis of the current distribution of archaic-like *HLA* alleles or haplotypes in present-day humans. The information on frequency distribution of *HLA* alleles across present-day human populations was collected from The Allele Frequency Net Database (AFND, http://www.allelefrequencies.net/, González-Galarza et al. 2015). Abi-Rached et al. (2011) reported that HLA-B*73 was the most frequently observed allele in west Asia, which was believed to be a site of admixture with Denisovans, and that this allele was rare or absent in other regions. However, the frequency of HLA-B*73 allele in west Asia (0.24 %) was lower than that in Europe and south Asia (Europe, 0.72 %; south Asia, 0.69 %; Table 1) although it is difficult to demonstrate that the difference is significant because of their low frequencies. This is inconsistent with the assumption that the highest frequency of HLA-B*73 in west Asia is an evidence of introgression of this allele (Abi-Rached et al. 2011). The frequency distribution of HLA-B*73 allele in Abi-Rached et al. (2011) appears to be estimated from the dataset derived from bone marrow donors, while the frequency distribution in the present study is based on the dataset in the AFND. Although the allele frequencies of HLA-B*73 are not shown in the previous study, in the present study, the observed number of individuals with HLA-B*73 is 3414 at a minimum in Europe, which is a population that the B*73 frequency is the highest, and in west Asia the number is 134 at a minimum (Table 1): These numbers are larger than those in the previous study (2677 in Europe and 128 in west Asia). Therefore, it is controversial whether the frequency of HLA-B*73 is the highest in west Asia.

**Table 1:** Allele frequencies of putative Denisovan-derived HLA-B*73 allele in The Allele Frequency Net Database

Population	Australia	Europe	North Africa	North-East Asia	Oceania	South Asia	South-East Asia	Sub-Saharan Africa	West Asia
HLA-B*73 ^a^									
No. of local populations	1	100	9	17	1	9	27	17	19
No. of chromosomes (2 *N*)	1782	945,368	2232	19,414	100	1698	171,600	4864	113,292
Observations	0	6828	6	30	0	12	11	14	268
Proportion	0.00 %	0.72 %	0.27 %	0.16 %	0.00 %	0.69 %	0.01 %	0.28 %	0.24 %

^a^2 digit and 4 digit data are merged (i. e., the first level of resolution)

Incidentally, Abi-Rached et al. (2011) suggested that the existence of B*73 in Africa may have resulted from the back migration from west Asia to Africa; however, it seems unlikely that such a low-frequency allele could have spread rapidly worldwide after the introgression. In addition, as far as we searched, the HLA-B*73 allele has not been observed in Melanesians, even though the Melanesian genome contains the highest proportion of Denisovan ancestry in present-day human populations (Reich et al. 2010; Qin and Stoneking 2015). Therefore, the global distribution of HLA-B*73 does not appear to support the introgression hypothesis.

With respect to Neanderthals, all six Neanderthal *HLA* class I alleles (the Vindija Neanderthals were heterozygotes at the *HLA-A*, *HLA-B*, and *HLA-C*) have been reported to be identical to their corresponding alleles in present-day humans (Abi-Rached et al. 2011). According to the AFND, some archaic-like alleles are observed in Africans (e.g., 6.1 % for HLA-B*51:01 in North Africa). Even if the admixture introduced archaic *HLA* alleles into modern humans, it is unlikely that all alleles of Neanderthals spread rapidly to modern humans. Although Temme et al. (2014) reported that a part of the amino acid residues of DPB1*04:01 was derived from Neanderthal introgression, Ding et al. (2014) argued against the possibility because DPB1*04:01 was frequently observed even in sub-Saharan Africans. Similar to DPB1*04:01, it is difficult to state with certainty that HLA-B*73 introgressed from Denisovans into early modern humans.

*HLA* allelic lineages have been maintained by balancing selection for lengthy periods in evolutionary time (Takahata 1990). To examine the genetic divergence among *HLA* alleles, nucleotide sequence data at three *HLA* class I loci (*HLA-A*, *HLA-B*, and *HLA-C*) and three class II loci (*HLA-DRB1*, *HLA-DQB1*, and *HLA-DPB1*) were retrieved from the NCBI database. The number of synonymous substitutions per synonymous site (*d*_S_) for all the pairs formed by *HLA* coding sequences (190 *HLA-A*, 308 *HLA-B*, 218 *HLA-C*, 64 *HLA-DRB1*, 11 *HLA-DQB1*, and 17 *HLA-DPB1*) were estimated by using the modified Nei-Gojobori model (Zhang et al. 1998) with Jukes–Cantor correction (Jukes and Cantor 1969) (*R* = 1.14 for class I and *R* = 1.04 for class II), in the same manner as Yasukochi and Satta (2013), in MEGA (Tamura et al. 2011). We found that the maximum *d*_S_ value (*d*_Smax_) of *HLA-B* alleles with HLA-B*73:01 (0.09) was similar to that of *HLA-DRB1* alleles (0.09) (Table 2). Previous studies showed the existence of two distinct allelic groups (groups A and B) at a single *HLA-DRB1* locus and the highly reduced genetic variation of group A allelic lineage in the common chimpanzee (Yasukochi and Satta 2014a; 2014b). These observations allow us to infer that the divergent HLA-B*73 can be maintained as a different *HLA-B* allelic group at a single *HLA-B* locus. In addition, the estimated selection coefficient (*s*) of *HLA-B* gene is the highest among the six classical *HLA* genes (Satta et al. 1994; Yasukochi and Satta 2013; dos Santos et al. 2015; Buhler et al. 2016). The larger the value of selection coefficient, the longer the persistence of *HLA* alleles (Takahata 1990). As mentioned earlier, chimpanzees also retained both *MHC-BI* and *MHC-BII* allelic lineages; therefore, it is plausible that all *MHC-BII* allelic lineages in Hominini have persisted for a very long evolutionary time through balancing selection.

**Table 2:** The *d*_S_ values of pairs formed by alleles in six *HLA* loci

*HLA* class	Locus	No. of allele	Length	*d*_Smean_^a^	*d*_Smax_^b^
Class I	*HLA-A*	190	1095 bp	0.04	0.07
	*HLA-B*	308	1086 bp	0.03	0.09
	*HLA-C*	218	1093 bp	0.03	0.06
Class II	*HLA-DRB1*	64	801 bp	0.04	0.09
	*HLA-DQB1*	11	786 bp^c^	0.07	0.11
	*HLA-DPB1*	17	777 bp^c^	0.02	0.04

^a^The mean number of synonymous substitutions per site

^b^The maximum number of synonymous substitutions per site

^c^Not whole coding sequence because of the insufficient number of sequences for calculation

Abi-Rached et al. (2011) stated that the strong LD between HLA-B*73 and HLA-C*15:05 in populations worldwide was a signature of introgression. However, it is not surprising that a low-frequent allele such as HLA-B*73 is in strong LD with some allele. It should be noted that the strong LD is not observed only between HLA-B*73 and HLA-C*15:05 alleles. For example, in the NCBI dbMHC database (http://www.ncbi.nlm.nih.gov/projects/gv/mhc, Meyer et al. 2007), we found that HLA-B*82, a rare allelic lineage in present-day humans, also exhibited strong LD with HLA-C*03:02 in Africans (Online Resource 3). Further, HLA-B*82 was also observed outside Africa (Online Resource 4). It is striking that the putative haplotype HLA-B*35:02-HLA-C*04:01 shows a quite similar pattern to the HLA-B*73-C*15:05 (Online Resource 5): The *HLA* class I genotype data of individuals with HLA-B*35:02 suggests that the LD of HLA-B*35:02 with HLA-C*04:01 is globally strong, but the LD in west Asia (South-West Asia in Online Resource 5) is somewhat weaker than that outside west Asia. In addition, C*15:05 is in LD with a variety of *HLA-B* alleles in many populations (AFND, http://www.allelefrequencies.net/, González-Galarza et al. 2015), implying that C*15:05 has been maintained in humans for a long time. Therefore, the strong LD between HLA-B*73 and HLA-C*15:05 does not appear to provide conclusive evidence against the recent admixture with archaic humans.

Abi-Rached et al. (2011) also reported that alleles consisting of all possible combinations of Denisovan *HLA-A* and *HLA-C* (i.e., possible *HLA-A-C* haplotypes that are combinations of HLA-A*11 and HLA-C*12:02 or C*15 alleles) are frequently distributed in Asia and Oceania and that modern humans acquired their haplotypes via admixture with Denisovans in the relatively recent past (Abi-Rached et al. 2011). However, according to the dbMHC database, these three alleles form haplotypes with a variety of *HLA-A* or *HLA-C* and *HLA-B* alleles, even in Africa (e.g., HLA-C*12:02 forms haplotypes with at least 30 *HLA-A* and 60 *HLA-B* alleles). In addition, four *HLA* alleles (HLA-B*07, B*51, C*07:02, and C*16:02), possible members of Neanderthal-like haplotypes (Abi-Rached et al. 2011), showed a similar trend as Denisovan-like haplotypes (e.g., HLA-C*07:02 forms haplotypes with at least 60 *HLA-A* and 130 *HLA-B* alleles). These results suggest that putative archaic alleles have already existed in modern humans prior to the archaic admixture. To confirm this, we further examined whether the HLA-A*11-HLA-C*12:02 and HLA-A*11-HLA-C*15 haplotypes were transmitted from Denisovans, by using a large integrated variant dataset from the 1000 Genomes Project (http://www.1000genomes.org/, The 1000 Genomes Project Consortium 2010). The dataset was obtained via the 1000 Genomes Browser (http://browser.1000genomes.org/). The genotype data at 20,937 biallelic sites throughout approximately 1.4 Mb genomic region from the *HLA-A* to *HLA-B* genes (via the *HLA-C*) was used for the analysis after excluding genotype data at monoallelic and multiallelic sites among 86 individuals [28 unrelated Japanese from Tokyo, 30 unrelated Han Chinese from Beijing, 11 unrelated Utah residents with Northern and Western European ancestry (CEU), and 17 unrelated Yoruba in Ibadan, Nigeria (YRI), whose DNA samples were used in Phase I to III of the International HapMap Project]: the data of individual who was a homozygote in at least one of *HLA* class I genes was excluded, and one of individuals who belong to the same pedigree was selected in the CEU and YRI. The genotype data of *HLA* class I loci in the 86 individuals were obtained from the report of de Bakker et al. (2006). If an individual is a heterozygote with the putative Denisovan and modern *HLA-A-C* haplotypes, heterozygous sites of such individuals are expected to be observed more frequently than those of individuals with modern haplotypes only. However, there was no distinctive difference in the number of heterozygous sites among all individuals examined (Online Resources 6 and 7). Furthermore, we generated phased haplotypes from the genotype data of 10 individuals described above and then constructed the phylogenetic tree on the basis of 19,334 biallelic SNPs after removing monoallelic/multiallelic SNPs and indels (Online Resource 8). The tree displayed two major clades, clades A and B. In the tree, archaic *HLA* haplotypes are expected to form a distinct clade from modern *HLA* haplotypes owing to large genetic divergence under the introgression hypothesis; however, *HLA* alleles of an individual with the archaic and modern *HLA* alleles did not always form a monophyletic clade, whereas the alleles of an individual with only modern haplotypes were sometimes assigned into different clades. These results indicate that putative archaic *HLA* haplotypes in modern humans have not actually introgressed via the recent admixture.

In conclusion, there is no clear evidence of introgression of the Denisovan-like *HLA* haplotypes into modern human genomes. Balancing selection at the *HLA-B* locus would have maintained the HLA-B*73 allelic lineage in the direct ancestors of modern humans for a long evolutionary time. A functional analysis of protein coded by HLA-B*73 may provide the reason for the long-term persistence of this allele.

## Electronic Supplementary Materials

Below is the link to the electronic supplementary material.

ESM 1 Figure S1. Maximum likelihood (ML) tree for genomic sequences around the *MHC-B* locus (ca. 8 kb). Six *HLA-C*sequences are used as the outgroup. The best-fit substitution model was estimated and the ML tree was constructed using the MEGA (Tamura et al. 2011). The reliability of monophyletic groups was assessed by the bootstrap analysis with 1000 replications and the bootstrap cut-off was set to 80 % (only bootstrap values over 80 % are shownin the trees). To obtain the ML tree, a nearest-neighbor-inter-change (NNI) search was applied. MHC-BI and MHC-BII represent allelic lineages identified in this study (see text). (PDF 260 kb)

ESM 2 Figure S2. The alignment of *MHC-BI* and *MHC-BII* alleles in Homininae. (PDF 262 kb)

ESM 3 Table S1. Genotype data of individuals with HLA-B*82 in the dbMHC database. (PDF 37.3 kb)

ESM 4 Table S2. Allele frequencies of HLA-B*82 allele in The Allele Frequency Net Database. (PDF 45.1 kb)

ESM 5 Table S3. Genotype data of individuals with HLA-B*35:02 in the dbMHC database. (PDF 72.8 kb)

ESM 6 Table S4. Comparison of the mean number of heterozygous sites in 20,937 biallelic sites across *HLA-A-C-B* region from 86 individuals (7 individuals with archaic-like *HLA-A-C* haplotypes and 79 individuals with modern *HLA-A-C* haplotypes only) in 1000 Genomes. (PDF 28.6 kb)

ESM 7 Figure S3. Distribution of heterozygous sites in 20,937 biallelic sites across *HLA-A-C-B* region (Chr. 6: 29,909,044-31,324,955) from 86 individuals (7 individuals with archaic-like *HLA-A-C* haplotypes and 79 individuals with modern *HLA-A-C* haplotypes only) in 1000 Genomes. Pink box represents the heterozygous site. Yellow box represents a *HLA* class I genomic region. The serial number in blue box indicates an individual with the archaic-like *HLA-A-C* haplotype. The serial number in green box indicates an individual with the modern *HLA-A-C* haplotypes only. The 1000 Genomes’ sample ID of each serial number is as follows: 1, NA11994; 2, NA12234; 3, NA12156; 4, NA18959; 5, NA18562; 6, NA18552; 7, NA18582; 8, NA11919; 9, NA12878; 10, NA07056; 11, NA10851; 12, NA11832; 13, NA12750; 14, NA12763; 15, NA12815; 16, NA18991; 17, NA18992; 18, NA18969; 19, NA18998; 20, NA18968; 21, NA18980; 22, NA18942; 23, NA18952; 24, NA18964; 25, NA18965; 26, NA18973; 27, NA18975; 28, NA18978; 29, NA18970; 30, NA18995; 31, NA18987; 32, NA18990; 33, NA18994; 34, NA18997; 35, NA18943; 36, NA19005; 37, NA18999; 38, NA19007; 39, NA18944; 40, NA18945; 41, NA18949; 42, NA18948; 43, NA18542; 44, NA18621; 45, NA18632; 46, NA18636; 47, NA18555; 48, NA18637; 49, NA18537; 50, NA18624; 51, NA18608; 52, NA18563; 53, NA18571; 54, NA18526; 55, NA18605; 56, NA18547; 57, NA18609; 58, NA18564; 59, NA18566; 60, NA18612; 61, NA18620; 62, NA18622; 63, NA18623; 64, NA18558; 65, NA18593; 66, NA18572; 67, NA18532; 68, NA18561; 69, NA18603; 70, NA18502; 71, NA18505; 72, NA18508; 73, NA18858; 74, NA18871; 75, NA18861; 76, NA19093; 77, NA19204; 78, NA19210; 79, NA19206; 80, NA19160; 81, NA19222; 82, NA19141; 83, NA19152; 84, NA19129; 85, NA19098; 86, NA19239. (PDF 2.32 mb)

ESM 8 Figure S4. Neighbor-joining tree with Kimura two-parameter model based on 19,334 SNPs without monoallelic/multiallelic and indel SNPs across *HLA-A-C-B* region from 86 individuals (Archaic-like *HLA-A-C* haplotypes from 7 individuals and Modern *HLA-A-C* haplotypes from 79 individuals) in 1000 Genomes. Only bootstrap values larger than 80 % are shown. Same color letters indicate *HLA* alleles in an individual. Yellow box represents *HLA* alleles of individual who was identified as a heterozygote of archaic and modern *HLA* alleles. (PDF 624 kb)
