# Supplementary material for: Elucidating the origin of HLA-B*73 allelic lineage: Did modern humans benefit by archaic introgression?
Source: Immunogenetics. 2016 Sep 30;69(1):63–7. doi: 10.1007/s00251-016-0952-8 (PMC5203853; doi:10.1007/s00251-016-0952-8)
Supplement: Supplementary file 1 — Figure S1. Maximum likelihood (ML) tree for genomic sequences around the MHC-B locus (ca. 8 kb). Six HLA-Csequences are used as the outgroup. The best-fit substitution model was estimated and the ML tree was constructed using the MEGA (Tamura et al. 2011). The reliability of monophyletic groups was assessed by the bootstrap analysis with 1000 replications and the bootstrap cut-off was set to 80 % (only bootstrap values over 80 % are shownin the trees). To obtain the ML tree, a nearest-neighbor-inter-change (NNI) search was applied. MHC-BI and MHC-BII represent allelic lineages identified in this study (see text). (PDF 260 kb) [file 251_2016_952_MOESM1_ESM.pdf]

Article title: Elucidating the origin of *HLA-B*73* allelic lineage: Did modern humans benefit by archaic introgression?; **Journal name:** Immunogenetics; **Authors names:** Yoshiki Yasukochi and Jun Ohashi; **Affiliation and e-mail address of the corresponding author:** Department of Human Genomics, Life Science Research Center, Mie University, 1577 Kurima-machiya, Tsu, Mie 514-8507, Japan. E-mail: hyasukou@proof.ocn.ne.jp

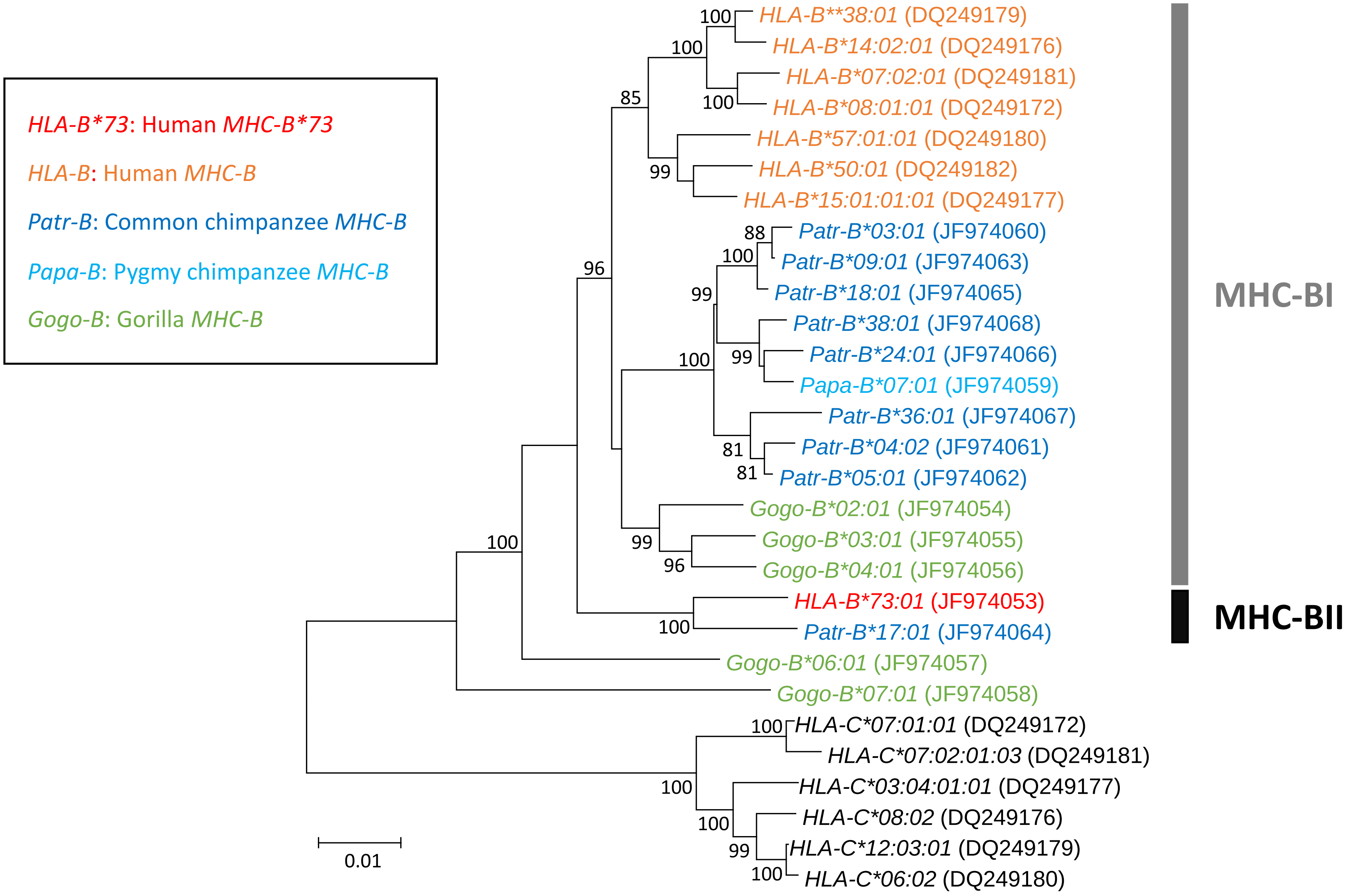

Figure S1 Maximum likelihood (ML) tree for genomic sequences around the *MHC-B* locus (ca. 8 kb). Six *HLA-C* sequences are used as the outgroup. The best-fit substitution model was estimated and the ML tree was constructed using the MEGA (Tamura et al. 2011). The reliability of monophyletic groups was assessed by the bootstrap analysis with 1,000 replications and the bootstrap cut-off was set to 80% (only bootstrap values over 80% are shown in the trees). To obtain the ML tree, a nearest-neighbor-inter-change (NNI) search was applied. MHC-BI and MHC-BII represent allelic lineages identified in this study (see text).
