# Supplementary figures and images for: Elucidating the origin of HLA-B*73 allelic lineage: Did modern humans benefit by archaic introgression?
Source: Immunogenetics. 2016 Sep 30;69(1):63–7. doi: 10.1007/s00251-016-0952-8 (PMC5203853; doi:10.1007/s00251-016-0952-8)

**Figure S2. The alignment of *MHC-BI* and *MHC-BII* alleles in Hominae**

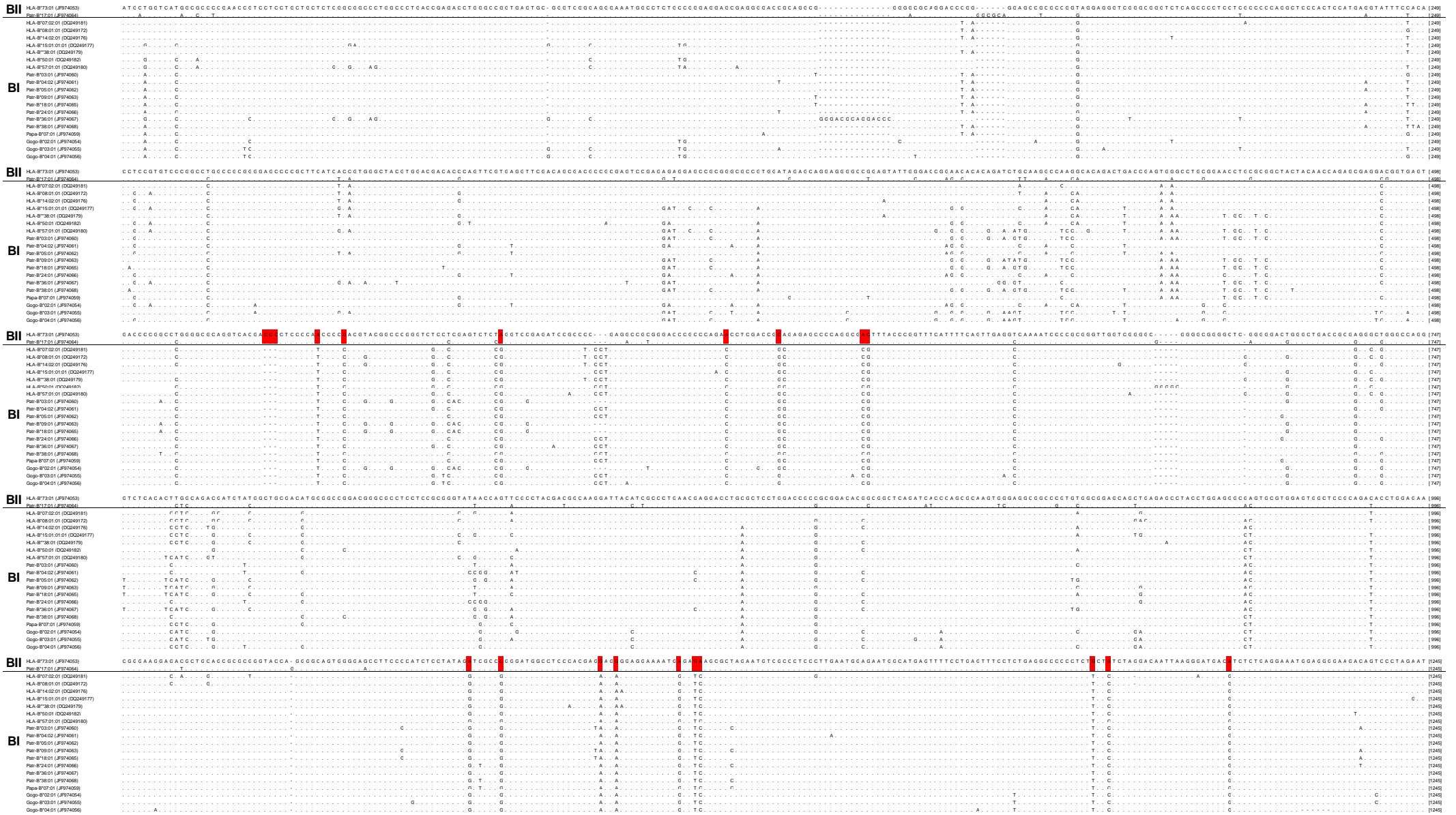

[illegible]

Supplement: Supplementary file 2 — Figure S2. The alignment of MHC-BI and MHC-BII alleles in Homininae. (PDF 262 kb) [file 251_2016_952_MOESM2_ESM.pdf]
