# Supplementary material for: Elucidating the origin of HLA-B*73 allelic lineage: Did modern humans benefit by archaic introgression?
Source: Immunogenetics. 2016 Sep 30;69(1):63–7. doi: 10.1007/s00251-016-0952-8 (PMC5203853; doi:10.1007/s00251-016-0952-8)
Supplement: Supplementary file 3 — Table S1. Genotype data of individuals with HLA-B*82 in the dbMHC database. (PDF 37.3 kb) [file 251_2016_952_MOESM3_ESM.pdf]

Article title: Elucidating the origin of *HLA-B*73* allelic lineage: Did modern humans benefit by archaic introgression?; **Journal name:** Immunogenetics; **Authors names:** Yoshiki Yasukochi and Jun Ohashi; **Affiliation and e-mail address of the corresponding author**: Department of Human Genomics, Life Science Research Center, Mie University, 1577 Kurima-machiya, Tsu, Mie 514-8507, Japan. **E-mail**: hyasukou@proof.ocn.ne.jp

Table S1. Genotype data of individuals with *HLA-B*82* in the dbMHC database

Pop. Area	HLA-A 1	HLA-A 2	HLA-B 1	HLA-B 2	HLA-C 1	HLA-C 2
Sub-Saharan Africa	*A*02:02:01:01*	*A*02:05:01*	*B*15:03:01:01*	*B*82:01*	*C*02:02*	*C*03:02*
Sub-Saharan Africa	*A*02:25*	*A*31:01:02:01*	*B*39:03*	*B*82:01*	*C*03:02*	*C*07:01*
Sub-Saharan Africa	*A*66:01:01*	*A*74:01:01*	*B*58:02*	*B*82:01*	*C*03:04*	*C*06:02:01:01*
Sub-Saharan Africa	*A*74:01:01*	*A*74:01:01*	*B*14:02*	*B*82:01*	*C*03:02*	*C*08:02:01:01*
Sub-Saharan Africa	*A*01:01*	*A*74:01:01*	*B*45:01:01*	*B*82:01*	*C*03:02*	*C*06:02:01:01*
Sub-Saharan Africa	*A*01:01*	*A*74:03*	*B*58:01:01:01*	*B*82:01*	*C*03:02*	*C*03:02*
Sub-Saharan Africa	*A*36:01*	*A*74:03*	*B*15:10:01*	*B*82:01*	*C*03:02*	*C*03:04:02*
Sub-Saharan Africa	*A*68:02:01:01*	*A*74:03*	*B*53:01*	*B*82:01*	*C*03:02*	*C*06:02:01:01*
Sub-Saharan Africa	*A*74:01:01*	*A*74:03*	*B*15:03:01:01*	*B*82:01*	*C*02:02*	*C*03:02*
Sub-Saharan Africa	*A*68:02:01:01*	*A*74:03*	*B*53:01*	*B*82:01*	*C*03:02*	*C*06:02:01:01*
Sub-Saharan Africa	*A*30:01*	*A*74:03*	*B*58:02*	*B*82:01*	*C*03:02*	*C*06:02:01:01*
Sub-Saharan Africa	*A*34:02:01*	*A*36:01*	*B*53:01*	*B*82:01*	*C*03:02*	*C*04:01*
Sub-Saharan Africa	*A*29:02*	*A*30:02*	*B*18:01*	*B*82:02:01*	*C*03:02*	*C*07:04*
Sub-Saharan Africa	*A*31:01:02:01*	*A*02:01:01*	*B*18:01*	*B*82:02:01*	*C*03:02*	*C*16:01*
Sub-Saharan Africa	*A*68:02:01:01*	*A*03:01:01*	*B*14:02*	*B*82:01*	*C*03:02*	*C*08:02:01:01*
Sub-Saharan Africa	*A*23:01:01*	*A*30:04:01*	*B*15:10:01*	*B*82:01*	–	–
Other	*A*01:01*	*A*23:01:01*	*B*52:01:02*	*B*82:01*	*C*08:02:01:01*	*C*16:01*
