# Supplementary material for: Elucidating the origin of HLA-B*73 allelic lineage: Did modern humans benefit by archaic introgression?
Source: Immunogenetics. 2016 Sep 30;69(1):63–7. doi: 10.1007/s00251-016-0952-8 (PMC5203853; doi:10.1007/s00251-016-0952-8)
Supplement: Supplementary file 4 — Table S2. Allele frequencies of HLA-B*82 allele in The Allele Frequency Net Database. (PDF 45.1 kb) [file 251_2016_952_MOESM4_ESM.pdf]

Article title: Elucidating the origin of *HLA-B*73* allelic lineage: Did modern humans benefit by archaic introgression?; **Journal name:** Immunogenetics; **Authors names:** Yoshiki Yasukochi* and Jun Ohashi; **Affiliation and e-mail address of the corresponding author**: Department of Human Genomics, Life Science Research Center, Mie University, 1577 Kurima-machiya, Tsu, Mie 514-8507, Japan. **E-mail**: hyasukou@proof.ocn.ne.jp

Table S2. Allele frequencies of *HLA-B*82* allele in The Allele Frequency Net Database

**Population**	**Australia**	**Europe**	**North Africa**	**North-East Asia**	**Oceania**	**South Asia**	**South-East Asia**	**Sub-Saharan Africa**	**Western Asia**
HLA-B*82									
No. of local populations	—	6	2	—	—	—	—	11	2
No. of chromosomes (2*N*)	—	87,566	564	—	—	—	—	3,696	1,424
Observations	—	86	13	—	—	—	—	26	2
Proportion	—	0.10%	2.30%	—	—	—	—	0.70%	0.13%
