# Supplementary material for: Elucidating the origin of HLA-B*73 allelic lineage: Did modern humans benefit by archaic introgression?
Source: Immunogenetics. 2016 Sep 30;69(1):63–7. doi: 10.1007/s00251-016-0952-8 (PMC5203853; doi:10.1007/s00251-016-0952-8)
Supplement: Supplementary file 5 — Table S3. Genotype data of individuals with HLA-B*35:02 in the dbMHC database. (PDF 72.8 kb) [file 251_2016_952_MOESM5_ESM.pdf]

Article title: Elucidating the origin of *HLA-B*73* allelic lineage: Did modern humans benefit by archaic introgression?; **Journal name:** Immunogenetics; **Authors names:** Yoshiki Yasukochi and Jun Ohashi; **Affiliation and e-mail address of the corresponding author**: Department of Human Genomics, Life Science Research Center, Mie University, 1577 Kurima-machiya, Tsu, Mie 514-8507, Japan. **E-mail**: hyasukou@proof.ocn.ne.jp

Table S3. Genotype data of individuals with *HLA-B*35:02* in the dbMHC database

Pop. Area	HLA-A 1	HLA-A 2	HLA-B 1	HLA-B 2	HLA-C 1	HLA-C 2
Europe	*A*02:05:01*	*A*11:01*	*B*35:02*	*B*50:01:01*	*C*04:01*	*C*06:02:01:01*
Europe	*A*02:01*	*A*24:05*	*B*35:02*	*B*44:02*	*C*04:01*	*C*05:01*
Europe	*A*24:02:01:01*	*A*31:01:02:01*	*B*18:01*	*B*35:02*	*C*04:01*	*C*07:01*
Europe	*A*01:01*	*A*02:01*	*B*15:01*	*B*35:02*	*C*03:04*	*C*06:02:01:01*
North-East Asia	*A*24:02:01:01*	*A*24:02:01:01*	*B*35:02*	*B*54:01:01*	*C*01:02*	*C*04:01*
North-East Asia	*A*03:01*	*A*24:02:01:01*	*B*35:02*	*B*39:01*	*C*04:01*	*C*12:03*
North-East Asia	*A*24:02:01:01*	*A*24:02:01:01*	*B*35:02*	*B*48:01:01*	*C*04:01*	*C*08:01*
North-East Asia	*A*24:02:01:01*	*A*24:02:01:01*	*B*35:02*	*B*40:02*	*C*03:04:01:01*	*C*04:01*
North-East Asia	*A*11:01*	*A*24:02:01:01*	*B*35:01*	*B*35:02*	*C*04:01*	*C*04:01*
South-East Asia	*A*24:02:01:01*	*A*24:07:01*	*B*15:13:01*	*B*35:02*	*C*04:01*	*C*08:01*
South-East Asia	*A*11:01*	*A*24:02*	*B*15:02:01*	*B*35:02*	*C*04:01*	*C*08:01*
South-East Asia	*A*29:01*	*A*33:03*	*B*07:05*	*B*35:02*	*C*04:03:01*	*C*06:02:01:01*
South-East Asia	*A*24:07:01*	*A*30:01*	*B*15:02:01*	*B*35:02*	*C*04:01*	*C*08:01*
South-West Asia	*A*02:01*	*A*02:11:01*	*B*35:02*	*B*51:07:01*	*C*04:01*	*C*04:01*
South-West Asia	*A*24:03*	*A*24:05*	*B*35:02*	*B*44:02*	*C*04:01*	*C*04:01*
South-West Asia	*A*03:02:01*	*A*11:01*	*B*35:02*	*B*35:20:01*	*C*04:01*	*C*05:01*
South-West Asia	*A*24:02:01:01*	*A*24:03*	*B*35:02*	*B*55:01*	*C*05:01*	*C*04:01*
South-West Asia	*A*01:01*	*A*32:01:01*	*B*35:02*	*B*40:02*	*C*02:02*	*C*04:07*
South-West Asia	*A*24:02:01:01*	*A*24:03*	*B*35:02*	*B*35:03:01*	*C*04:01*	*C*04:01*
South-West Asia	*A*02:05:01*	*A*24:02:01:01*	*B*35:02*	*B*58:01:01:01*	*C*04:01*	*C*07:01*
South-West Asia	*A*01:01*	*A*02:01*	*B*27:05*	*B*35:02*	*C*02:02*	*C*04:07*
South-West Asia	*A*03:01*	*A*24:02:01:01*	*B*14:04*	*B*35:02*	*C*04:01*	*C*08:02:01:01*

South-West Asia	*A*02:19*	*A*24:13:01*	*B*18:01*	*B*35:02*	*C*04:07*	*C*07:01*
South-West Asia	*A*02:01*	*A*02:05:01*	*B*15:08:01*	*B*35:02*	*C*01:02*	*C*04:01*
Sub-Saharan Africa	*A*24:02*	*A*32:01:01*	*B*35:02*	*B*81:01*	*C*04:01*	*C*04:01*
Sub-Saharan Africa	*A*24:02*	*A*29:01*	*B*35:02*	*B*44:03*	*C*04:01*	*C*04:01*
Sub-Saharan Africa	*A*23:01:01*	*A*31:01:02:01*	*B*35:02*	*B*44:03*	*C*04:01*	*C*04:01*
Sub-Saharan Africa	*A*68:02:01:01*	*A*68:02:01:01*	*B*15:10:01*	*B*35:02*	*C*03:04:02*	*C*04:01*
Sub-Saharan Africa	*A*02:01*	*A*30:02*	*B*08:01*	*B*35:02*	*C*04:01*	*C*07:01*
Sub-Saharan Africa	*A*24:02*	*A*24:02*	*B*35:01*	*B*35:02*	*C*04:01*	*C*07:01*
Sub-Saharan Africa	*A*24:02*	*A*29:02*	*B*14:02*	*B*35:02*	*C*04:01*	*C*08:02:01:01*
Sub-Saharan Africa	*A*02:01*	*A*74:01:01*	*B*08:01*	*B*35:02*	*C*04:01*	*C*07:01*
Sub-Saharan Africa	*A*02:01*	*A*25:01*	*B*27:05*	*B*35:02*	*C*02:02*	*C*04:01*
North America	*A*24:02*	*A*68:02:01:01*	*B*14:02*	*B*35:02*	*C*04:01*	*C*08:02:01:01*
Other[a]	*A*24:02:01:01*	*A*24:02:01:01*	*B*35:02*	*B*38:01:01*	*C*04:01*	*C*12:03*
Other[a]	*A*02:02:01:01*	*A*30:01*	*B*35:02*	*B*42:01:01*	*C*04:01*	*C*17:01:01:01*
Other[b]	*A*03:01*	*A*24:02*	*B*35:02*	*B*35:12:01*	*C*04:01*	*C*06:02:01:01*
Other[b]	*A*24:02*	*A*31:01:02:01*	*B*35:02*	*B*35:17:01*	*C*04:01*	*C*07:01*
Other[a]	*A*01:01*	*A*25:01*	*B*08:01*	*B*35:02*	*C*04:01*	*C*07:01*

[a] local population is Brazilian (Af Eu)
[b] local population is North America (Hi)
