# Supplementary material for: Elucidating the origin of HLA-B*73 allelic lineage: Did modern humans benefit by archaic introgression?
Source: Immunogenetics. 2016 Sep 30;69(1):63–7. doi: 10.1007/s00251-016-0952-8 (PMC5203853; doi:10.1007/s00251-016-0952-8)
Supplement: Supplementary file 6 — Table S4. Comparison of the mean number of heterozygous sites in 20,937 biallelic sites across HLA-A-C-B region from 86 individuals (7 individuals with archaic-like HLA-A-C haplotypes and 79 individuals with modern HLA-A-C haplotypes only) in 1000 Genomes. (PDF 28.6 kb) [file 251_2016_952_MOESM6_ESM.pdf]

Article title: Elucidating the origin of *HLA-B*73* allelic lineage: Did modern humans benefit by archaic introgression?; **Journal name:** Immunogenetics; **Authors names:** Yoshiki Yasukochi and Jun Ohashi; **Affiliation and e-mail address of the corresponding author**: Department of Human Genomics, Life Science Research Center, Mie University, 1577 Kurima-machiya, Tsu, Mie 514-8507, Japan. E-mail: hyasukou@proof.ocn.ne.jp

Table S4. Comparison of the mean number of heterozygous sites in 20,937 biallelic sites across *HLA-A-C-B* region from 86 individuals (7 individuals with archaic-like *HLA-A-C* haplotypes and 79 individuals with modern *HLA-A-C* haplotypes only) in 1000 Genomes

	**Homozygous site**	**Heterozygous site**
Genotype of individuals with "Archaic" *HLA-A-C* haplotypes[a]	16,671 ± 386	4,266 ± 386
Genotype of individuals with "Modern" *HLA-A-C* haplotypes only	16,337 ± 79	4,600 ± 79

[a] Possible Denisovan *HLA-A-C* haplotype that is any one of following two possible combinations: *HLA-A*11-C*12:02* and *HLA-A*11-C*15*
