# Supplementary material for: Elucidating the origin of HLA-B*73 allelic lineage: Did modern humans benefit by archaic introgression?
Source: Immunogenetics. 2016 Sep 30;69(1):63–7. doi: 10.1007/s00251-016-0952-8 (PMC5203853; doi:10.1007/s00251-016-0952-8)
Supplement: Supplementary file 7 — Figure S3. Distribution of heterozygous sites in 20,937 biallelic sites across HLA-A-C-B region (Chr. 6: 29,909,044-31,324,955) from 86 individuals (7 individuals with archaic-like HLA-A-C haplotypes and 79 individuals with modern HLA-A-C haplotypes only) in 1000 Genomes. Pink box represents the heterozygous site. Yellow box represents a HLA class I genomic region. The serial number in blue box indicates an individual with the archaic-like HLA-A-C haplotype. The serial number in green box indicates an individual with the modern HLA-A-C haplotypes only. The 1000 Genomes’ sample ID of each serial number is as follows: 1, NA11994; 2, NA12234; 3, NA12156; 4, NA18959; 5, NA18562; 6, NA18552; 7, NA18582; 8, NA11919; 9, NA12878; 10, NA07056; 11, NA10851; 12, NA11832; 13, NA12750; 14, NA12763; 15, NA12815; 16, NA18991; 17, NA18992; 18, NA18969; 19, NA18998; 20, NA18968; 21, NA18980; 22, NA18942; 23, NA18952; 24, NA18964; 25, NA18965; 26, NA18973; 27, NA18975; 28, NA18978; 29, NA18970; 30, NA18995; 31, NA18987; 32, NA18990; 33, NA18994; 34, NA18997; 35, NA18943; 36, NA19005; 37, NA18999; 38, NA19007; 39, NA18944; 40, NA18945; 41, NA18949; 42, NA18948; 43, NA18542; 44, NA18621; 45, NA18632; 46, NA18636; 47, NA18555; 48, NA18637; 49, NA18537; 50, NA18624; 51, NA18608; 52, NA18563; 53, NA18571; 54, NA18526; 55, NA18605; 56, NA18547; 57, NA18609; 58, NA18564; 59, NA18566; 60, NA18612; 61, NA18620; 62, NA18622; 63, NA18623; 64, NA18558; 65, NA18593; 66, NA18572; 67, NA18532; 68, NA18561; 69, NA18603; 70, NA18502; 71, NA18505; 72, NA18508; 73, NA18858; 74, NA18871; 75, NA18861; 76, NA19093; 77, NA19204; 78, NA19210; 79, NA19206; 80, NA19160; 81, NA19222; 82, NA19141; 83, NA19152; 84, NA19129; 85, NA19098; 86, NA19239. (PDF 2.32 mb) [file 251_2016_952_MOESM7_ESM.pdf]

Article title: Elucidating the origin of *HLA-B*73* allelic lineage: Did modern humans benefit by archaic introgression?; **Journal name:** Immunogenetics; **Authors names:** Yoshiki Yasukochi and Jun Ohashi; **Affiliation and e-mail address of the corresponding author:** Department of Human Genomics, Life Science Research Center, Mie University, 1577 Kurima-machiya, Tsu, Mie 514-8507, Japan. E-mail: hyasukou@proof.ocn.ne.jp

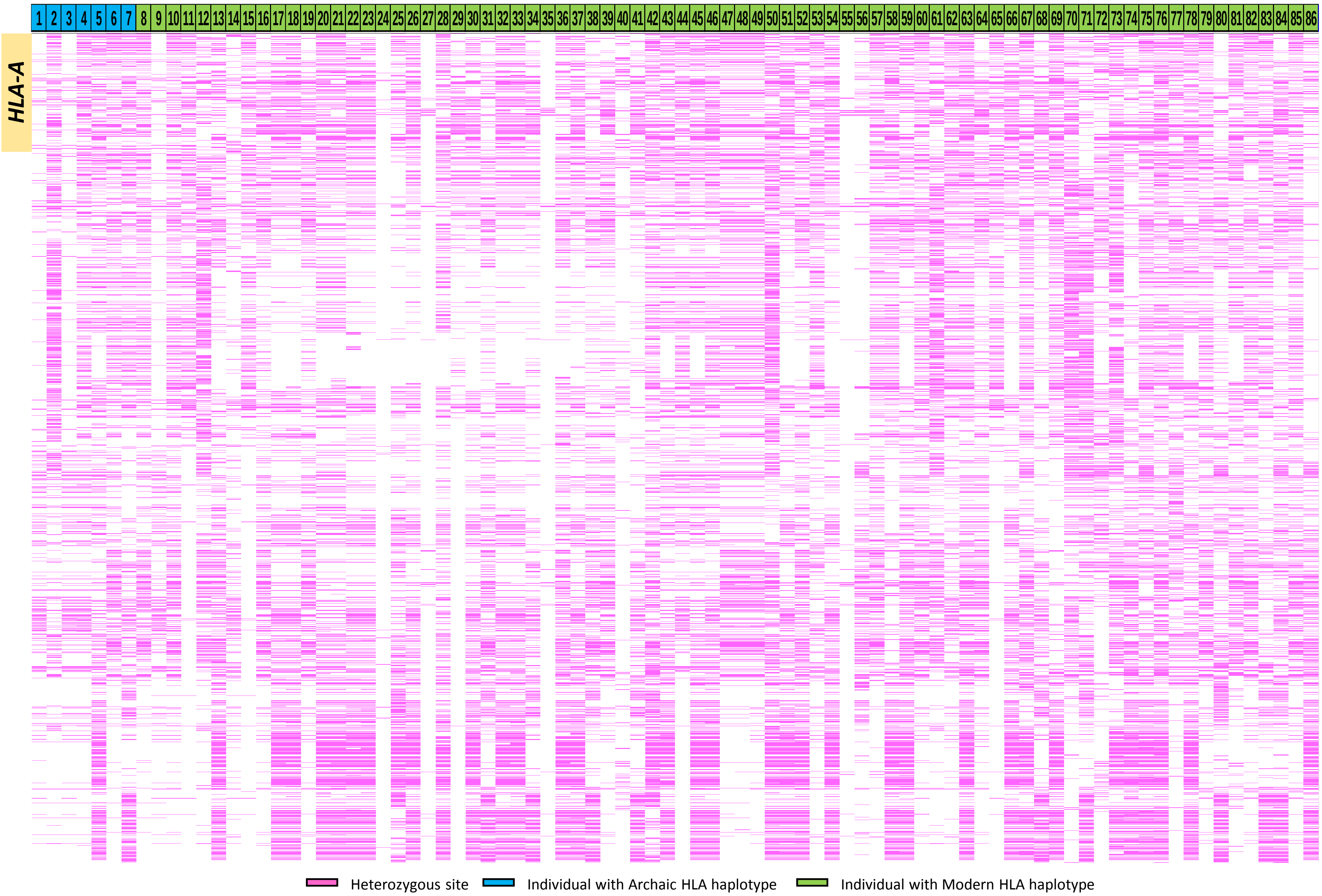

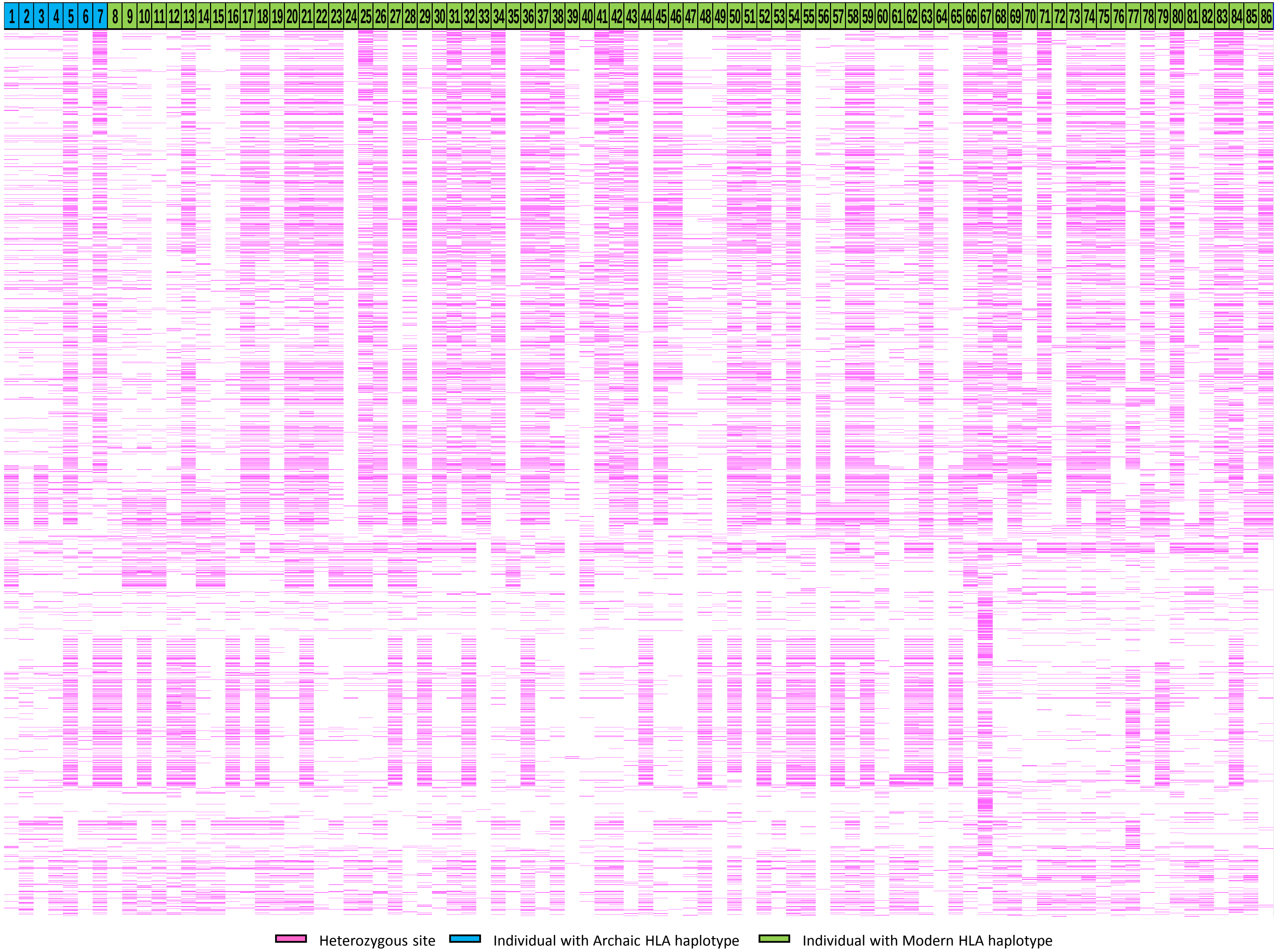
1 2 3 4 5 6 7 8 9 10 11 12 13 14 15 16 17 18 19 20 21 22 23 24 25 26 27 28 29 30 31 32 33 34 35 36 37 38 39 40 41 42 43 44 45 46 47 48 49 50 51 52 53 54 55 56 57 58 59 60 61 62 63 64 65 66 67 68 69 70 71 72 73 74 75 76 77 78 79 80 81 82 83 84 85 86
Heterozygous site
Individual with Archaic HLA haplotype
Individual with Modern HLA haplotype

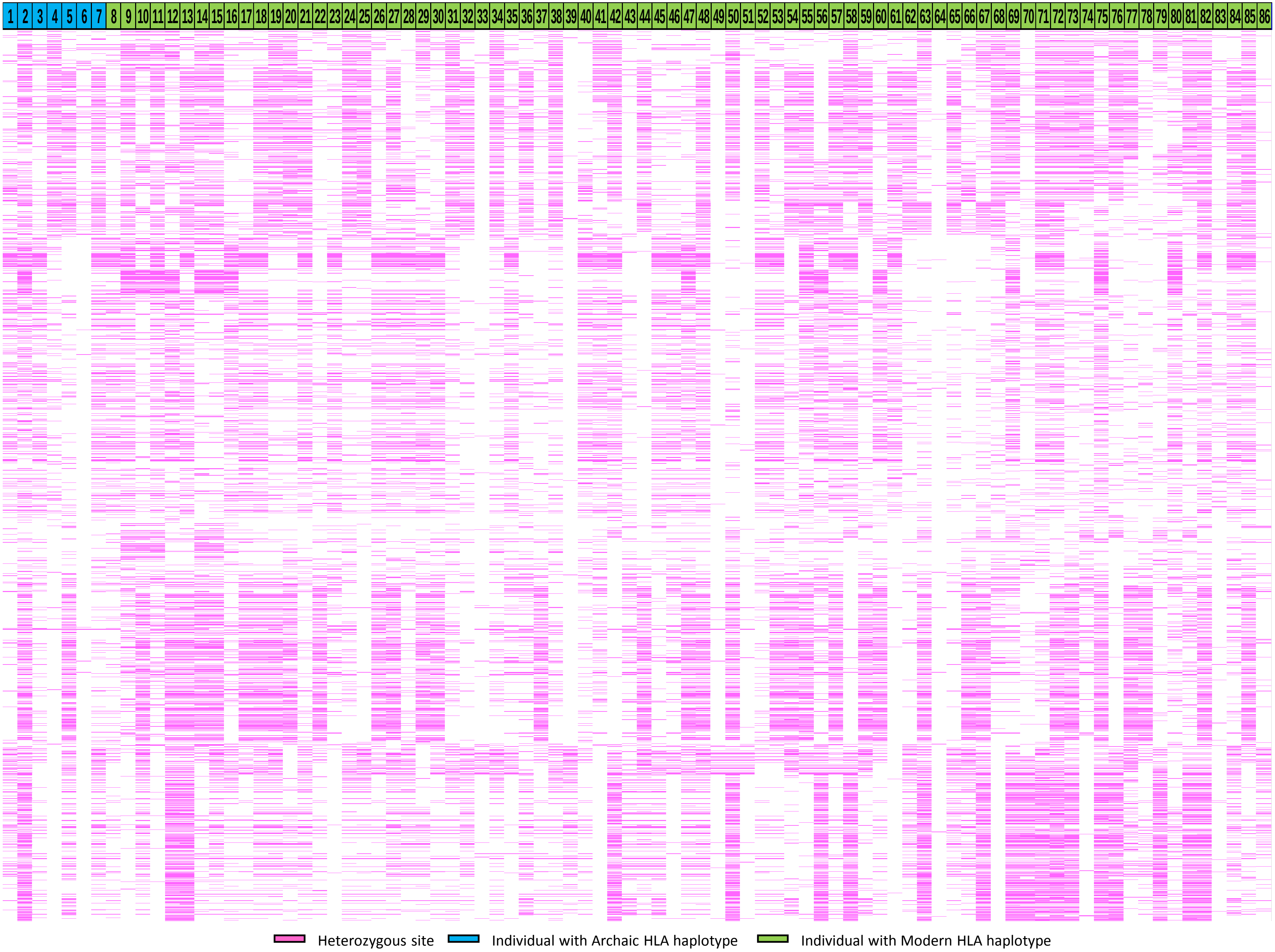
1 2 3 4 5 6 7 8 9 10 11 12 13 14 15 16 17 18 19 20 21 22 23 24 25 26 27 28 29 30 31 32 33 34 35 36 37 38 39 40 41 42 43 44 45 46 47 48 49 50 51 52 53 54 55 56 57 58 59 60 61 62 63 64 65 66 67 68 69 70 71 72 73 74 75 76 77 78 79 80 81 82 83 84 85 86
Heterozygous site
Individual with Archaic HLA haplotype
Individual with Modern HLA haplotype

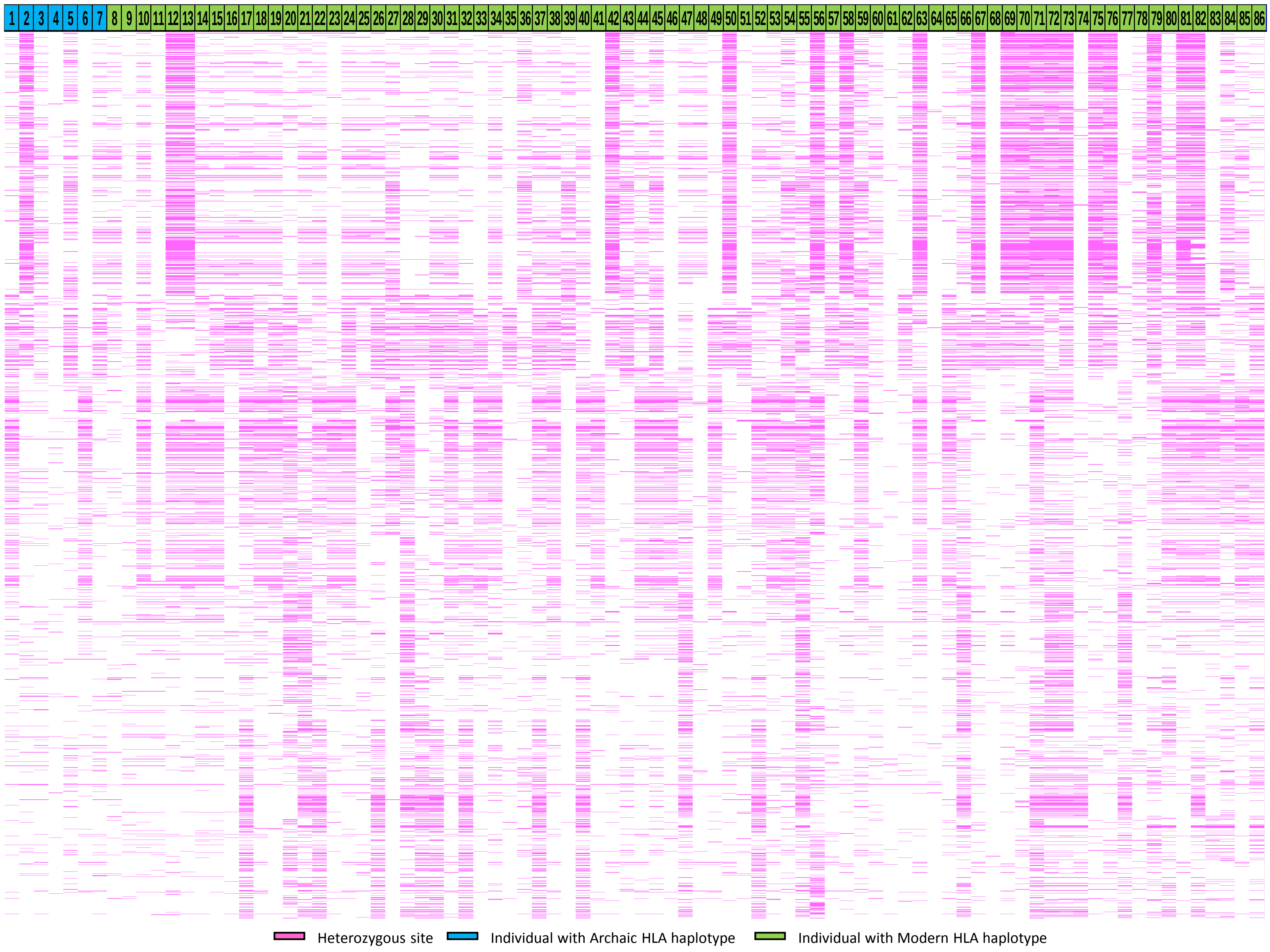
1 2 3 4 5 6 7 8 9 10 11 12 13 14 15 16 17 18 19 20 21 22 23 24 25 26 27 28 29 30 31 32 33 34 35 36 37 38 39 40 41 42 43 44 45 46 47 48 49 50 51 52 53 54 55 56 57 58 59 60 61 62 63 64 65 66 67 68 69 70 71 72 73 74 75 76 77 78 79 80 81 82 83 84 85 86
Heterozygous site
Individual with Archaic HLA haplotype
Individual with Modern HLA haplotype

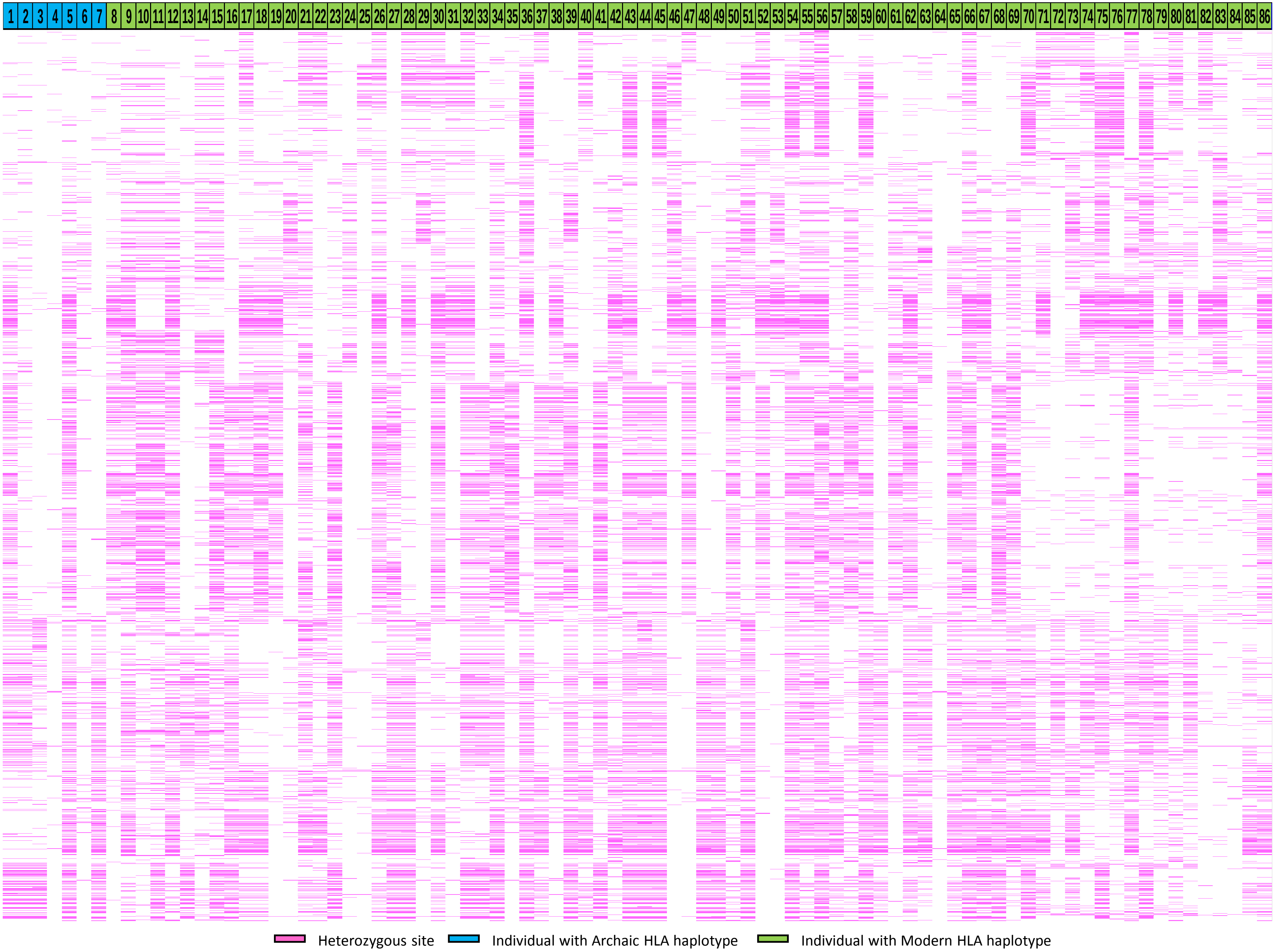
1 2 3 4 5 6 7 8 9 10 11 12 13 14 15 16 17 18 19 20 21 22 23 24 25 26 27 28 29 30 31 32 33 34 35 36 37 38 39 40 41 42 43 44 45 46 47 48 49 50 51 52 53 54 55 56 57 58 59 60 61 62 63 64 65 66 67 68 69 70 71 72 73 74 75 76 77 78 79 80 81 82 83 84 85 86
Heterozygous site
Individual with Archaic HLA haplotype
Individual with Modern HLA haplotype

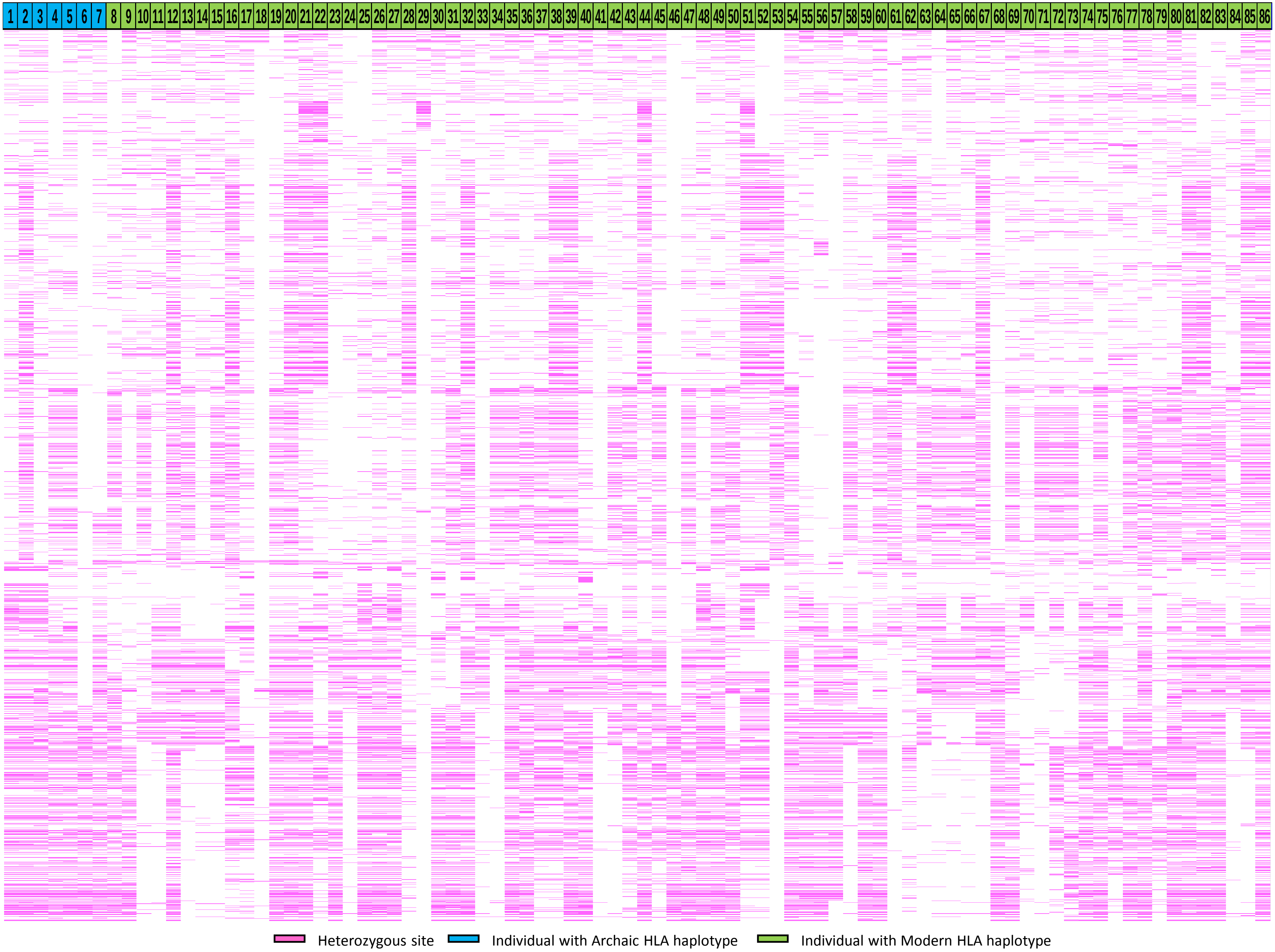
1 2 3 4 5 6 7 8 9 10 11 12 13 14 15 16 17 18 19 20 21 22 23 24 25 26 27 28 29 30 31 32 33 34 35 36 37 38 39 40 41 42 43 44 45 46 47 48 49 50 51 52 53 54 55 56 57 58 59 60 61 62 63 64 65 66 67 68 69 70 71 72 73 74 75 76 77 78 79 80 81 82 83 84 85 86
Heterozygous site
Individual with Archaic HLA haplotype
Individual with Modern HLA haplotype

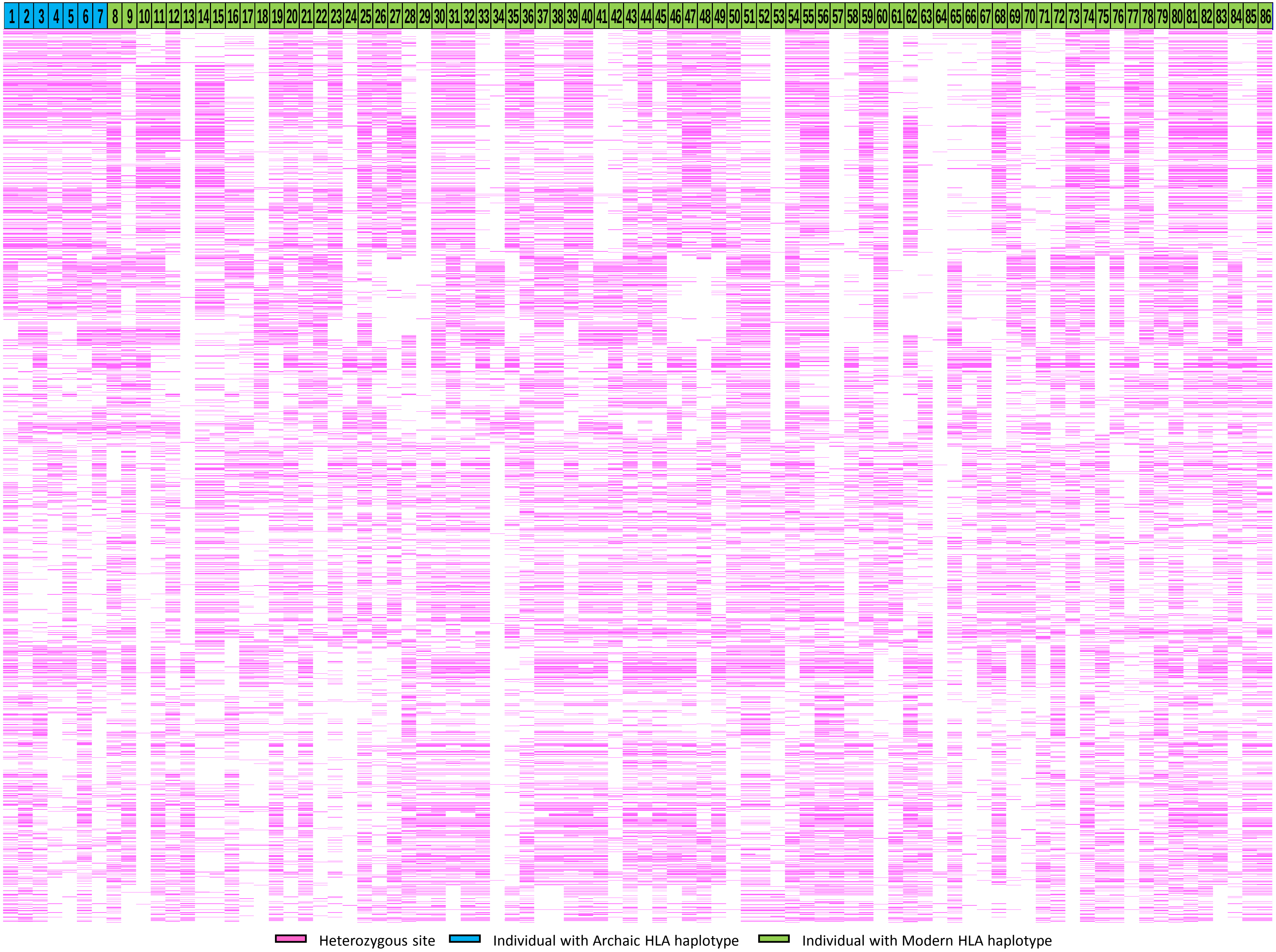
1 2 3 4 5 6 7 8 9 10 11 12 13 14 15 16 17 18 19 20 21 22 23 24 25 26 27 28 29 30 31 32 33 34 35 36 37 38 39 40 41 42 43 44 45 46 47 48 49 50 51 52 53 54 55 56 57 58 59 60 61 62 63 64 65 66 67 68 69 70 71 72 73 74 75 76 77 78 79 80 81 82 83 84 85 86
Heterozygous site
Individual with Archaic HLA haplotype
Individual with Modern HLA haplotype

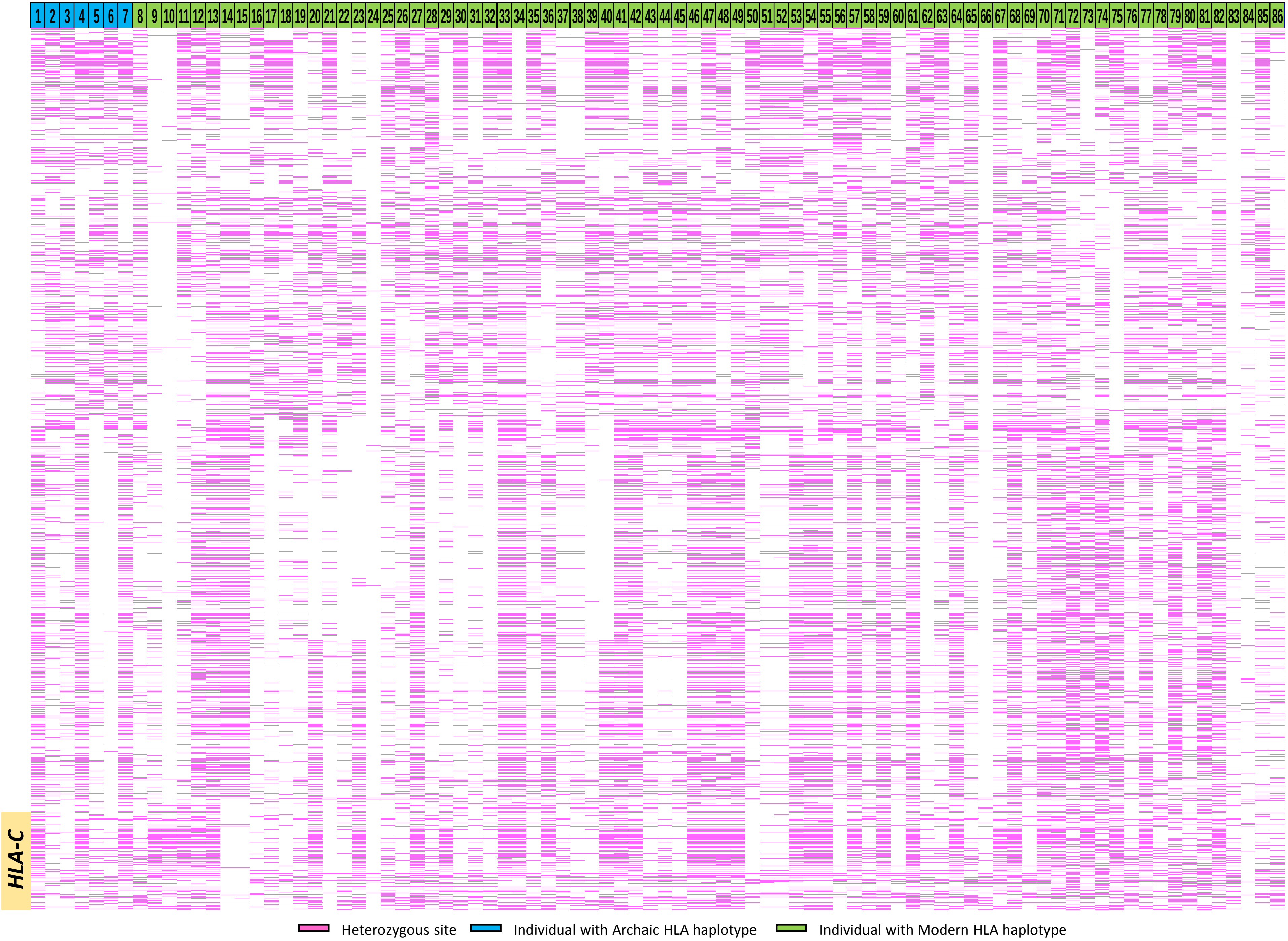
1 2 3 4 5 6 7 8 9 10 11 12 13 14 15 16 17 18 19 20 21 22 23 24 25 26 27 28 29 30 31 32 33 34 35 36 37 38 39 40 41 42 43 44 45 46 47 48 49 50 51 52 53 54 55 56 57 58 59 60 61 62 63 64 65 66 67 68 69 70 71 72 73 74 75 76 77 78 79 80 81 82 83 84 85 86
HLA-C
Heterozygous site
Individual with Archaic HLA haplotype
Individual with Modern HLA haplotype

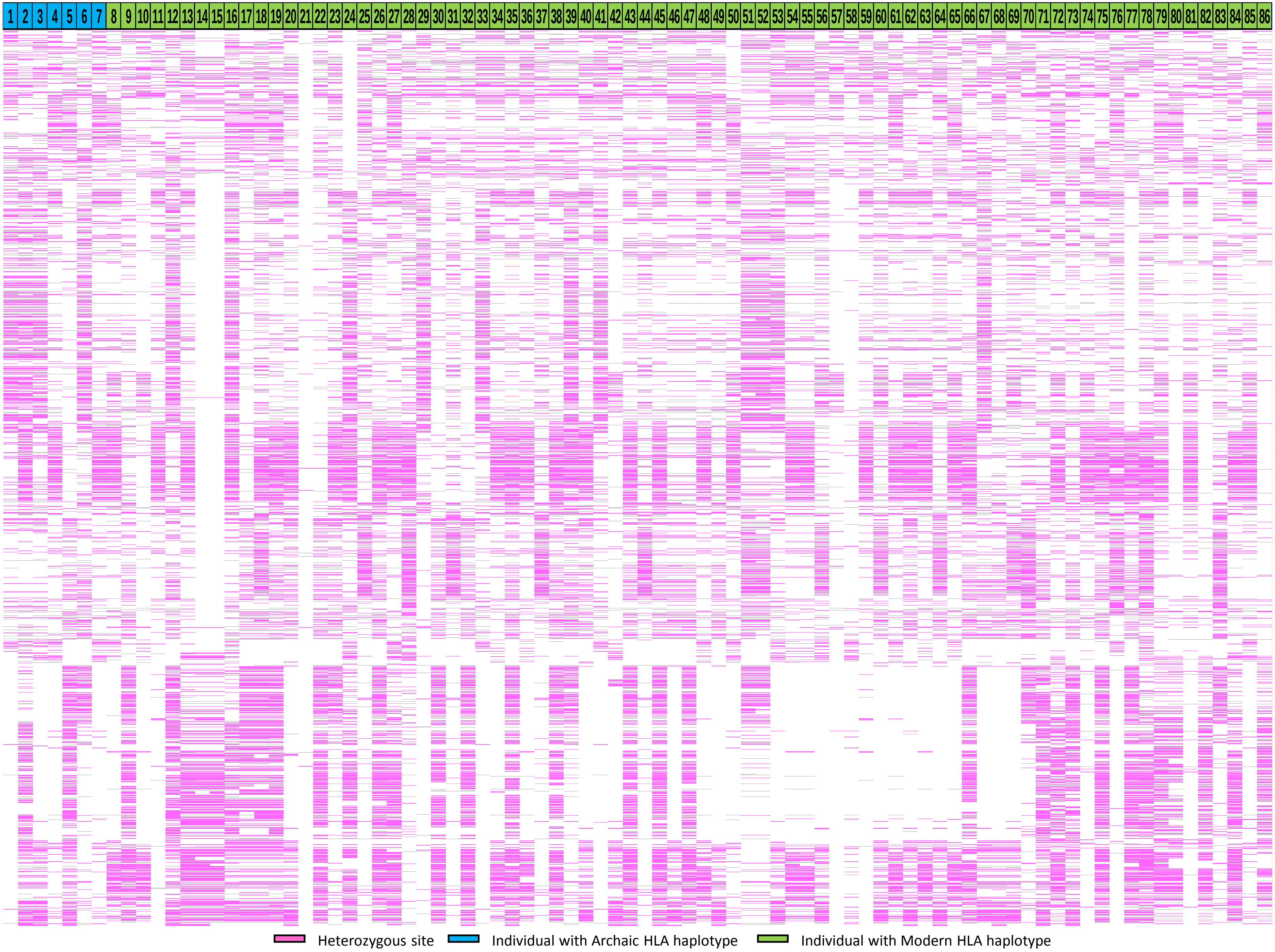
1 2 3 4 5 6 7 8 9 10 11 12 13 14 15 16 17 18 19 20 21 22 23 24 25 26 27 28 29 30 31 32 33 34 35 36 37 38 39 40 41 42 43 44 45 46 47 48 49 50 51 52 53 54 55 56 57 58 59 60 61 62 63 64 65 66 67 68 69 70 71 72 73 74 75 76 77 78 79 80 81 82 83 84 85 86
Heterozygous site
Individual with Archaic HLA haplotype
Individual with Modern HLA haplotype

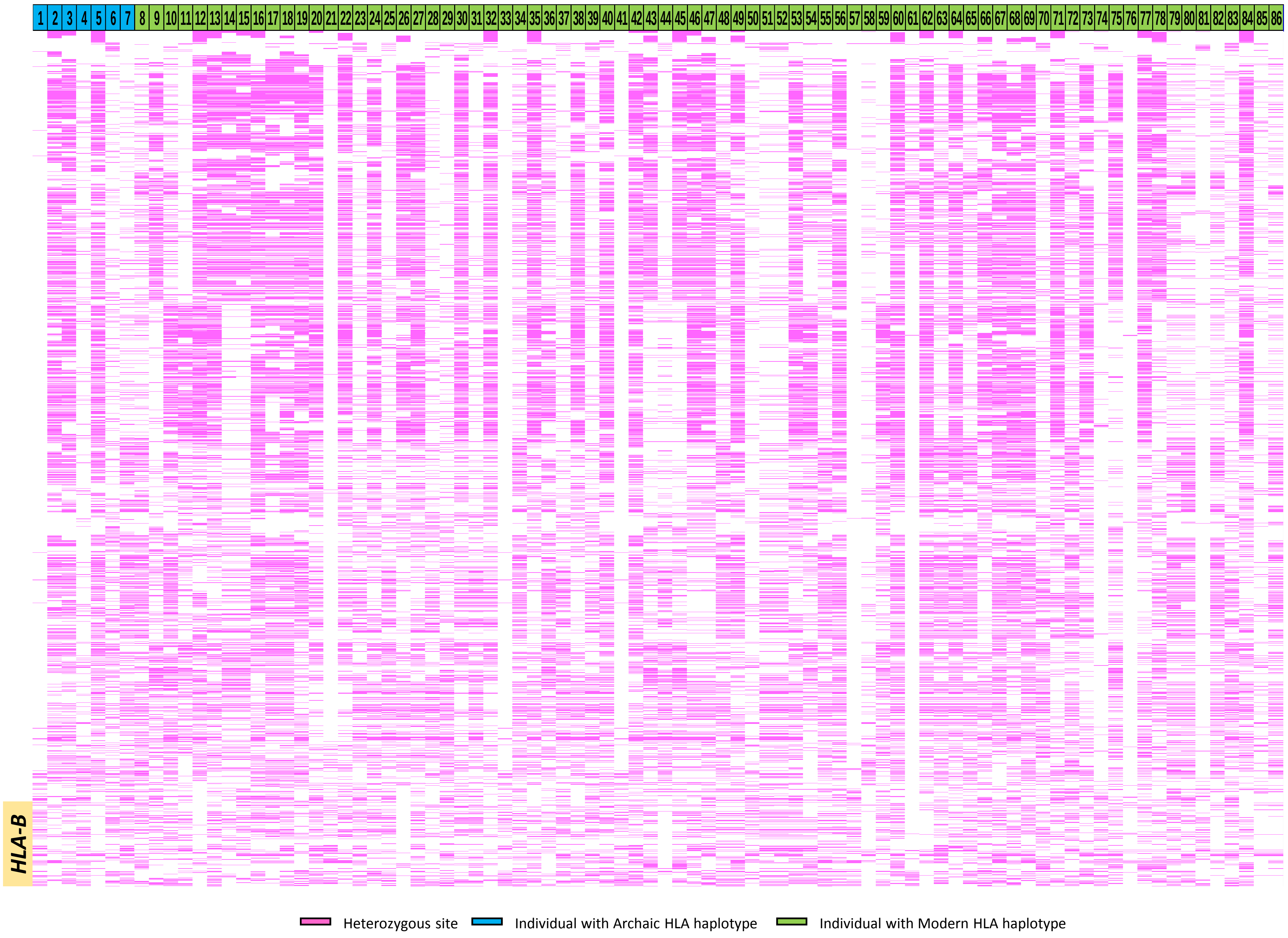
1 2 3 4 5 6 7 8 9 10 11 12 13 14 15 16 17 18 19 20 21 22 23 24 25 26 27 28 29 30 31 32 33 34 35 36 37 38 39 40 41 42 43 44 45 46 47 48 49 50 51 52 53 54 55 56 57 58 59 60 61 62 63 64 65 66 67 68 69 70 71 72 73 74 75 76 77 78 79 80 81 82 83 84 85 86
HLA-B
Heterozygous site
Individual with Archaic HLA haplotype
Individual with Modern HLA haplotype

Figure S3 Distribution of heterozygous sites in 20,937 biallelic sites across *HLA-A-C-B* region (Chr. 6: 29,909,044 - 31,324,955) from 86 individuals (7 individuals with archaic-like *HLA-A-C* haplotypes and 79 individuals with modern *HLA-A-C* haplotypes only) in 1000 Genomes. Pink box represents the heterozygous site. Yellow box represents a *HLA* class I genomic region. The serial number in blue box indicates an individual with the archaic-like *HLA-A-C* haplotype. The serial number in green box indicates an individual with the modern *HLA-A-C* haplotypes only. The 1000 Genomes' sample ID of each serial number is as follows: 1, NA11994; 2, NA12234; 3, NA12156; 4, NA18959; 5, NA18562; 6, NA18552; 7, NA18582; 8, NA11919; 9, NA12878; 10, NA07056; 11, NA10851; 12, NA11832; 13, NA12750; 14, NA12763; 15, NA12815; 16, NA18991; 17, NA18992; 18, NA18969; 19, NA18998; 20, NA18968; 21, NA18980; 22, NA18942; 23, NA18952; 24, NA18964; 25, NA18965; 26, NA18973; 27, NA18975; 28, NA18978; 29, NA18970; 30, NA18995; 31, NA18987; 32, NA18990; 33, NA18994; 34, NA18997; 35, NA18943; 36, NA19005; 37, NA18999; 38, NA19007; 39, NA18944; 40, NA18945; 41, NA18949; 42, NA18948; 43, NA18542; 44, NA18621; 45, NA18632; 46, NA18636; 47, NA18555; 48, NA18637; 49, NA18537; 50, NA18624; 51, NA18608; 52, NA18563; 53, NA18571; 54, NA18526; 55, NA18605; 56, NA18547; 57, NA18609; 58, NA18564; 59, NA18566; 60, NA18612; 61, NA18620; 62, NA18622; 63, NA18623; 64, NA18558; 65, NA18593; 66, NA18572; 67, NA18532; 68, NA18561; 69, NA18603; 70, NA18502; 71, NA18505; 72, NA18508; 73, NA18858; 74, NA18871; 75, NA18861; 76, NA19093; 77, NA19204; 78, NA19210; 79, NA19206; 80, NA19160; 81, NA19222; 82, NA19141; 83, NA19152; 84, NA19129; 85, NA19098; 86, NA19239.
