# Supplementary material for: Elucidating the origin of HLA-B*73 allelic lineage: Did modern humans benefit by archaic introgression?
Source: Immunogenetics. 2016 Sep 30;69(1):63–7. doi: 10.1007/s00251-016-0952-8 (PMC5203853; doi:10.1007/s00251-016-0952-8)
Supplement: Supplementary file 8 — Figure S4. Neighbor-joining tree with Kimura two-parameter model based on 19,334 SNPs without monoallelic/multiallelic and indel SNPs across HLA-A-C-B region from 86 individuals (Archaic-like HLA-A-C haplotypes from 7 individuals and Modern HLA-A-C haplotypes from 79 individuals) in 1000 Genomes. Only bootstrap values larger than 80 % are shown. Same color letters indicate HLA alleles in an individual. Yellow box represents HLA alleles of individual who was identified as a heterozygote of archaic and modern HLA alleles. (PDF 624 kb) [file 251_2016_952_MOESM8_ESM.pdf]

Article title: Elucidating the origin of *HLA-B*73* allelic lineage: Did modern humans benefit by archaic introgression?; **Journal name:** Immunogenetics; **Authors names:** Yoshiki Yasukochi and Jun Ohashi; **Affiliation and e-mail address of the corresponding author:** Department of Human Genomics, Life Science Research Center, Mie University 1577 Kurima-machiya, Tsu, Mie 514-8507, Japan. E-mail: hyasukou@proof.ocn.ne.jp

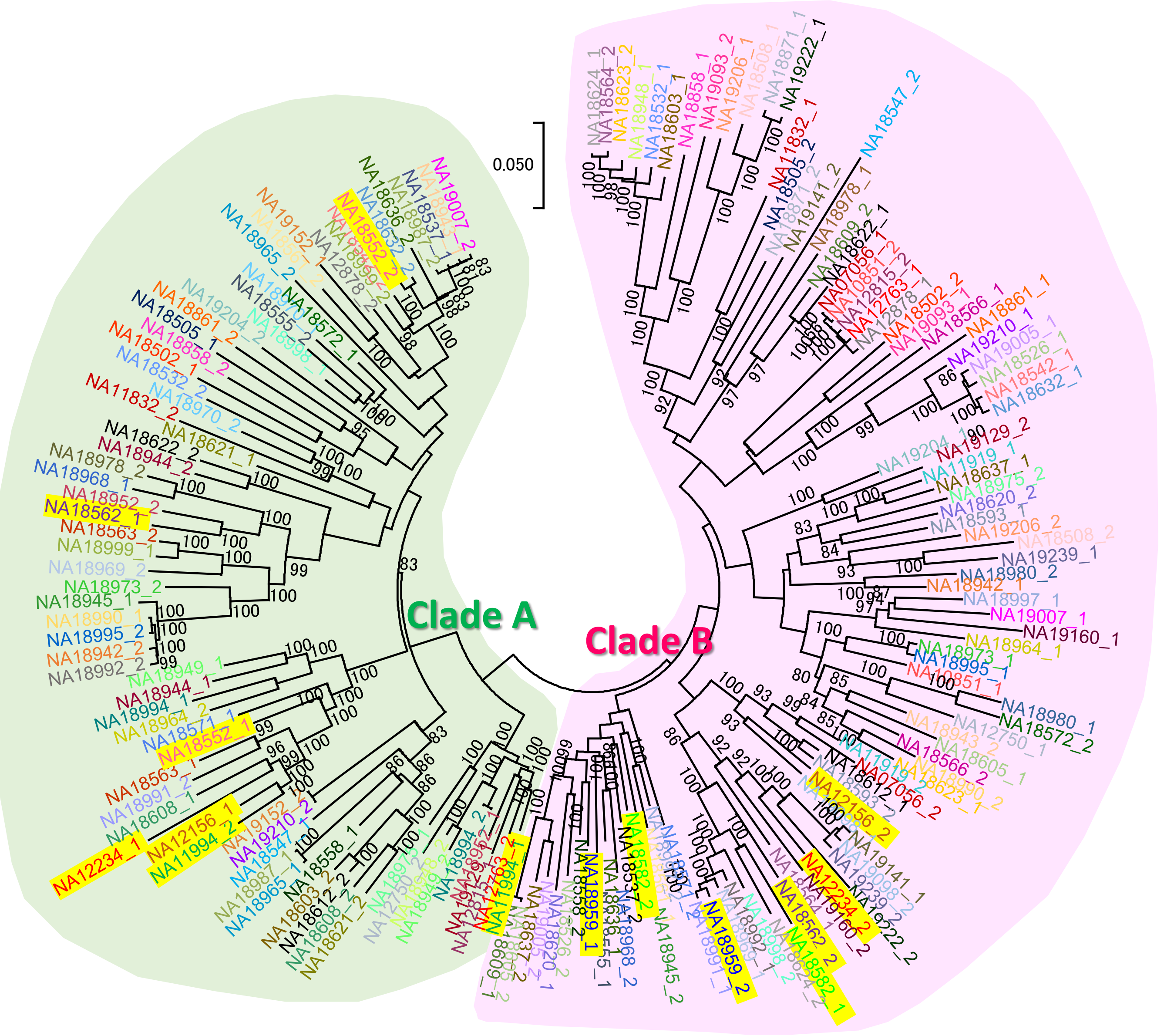

Figure S4 Neighbor-joining tree with Kimura two-parameter model based on 19,334 SNPs without monoallelic/multiallelic and indel SNPs across *HLA-A-C-B* region from 86 individuals (Archaic-like *HLA-A-C* haplotypes from 7 individuals and Modern *HLA-A-C* haplotypes from 79 individuals) in 1000 Genomes. Only bootstrap values larger than 80% are shown. Same color letters indicate *HLA* alleles in an individual. Yellow box represents *HLA* alleles of individual who was identified as a heterozygote of archaic and modern *HLA* alleles.
